# Sex differences in ventricular arrhythmia, atrial fibrillation and atrioventricular block complicating acute myocardial infarction

**DOI:** 10.3389/fcvm.2023.1217525

**Published:** 2023-10-19

**Authors:** Hilmi Alnsasra, Gal Tsaban, Jean Marc Weinstein, Mhamad Nasasra, Tal Ovdat, Roy Beigel, Katia Orvin, Moti Haim

**Affiliations:** ^1^ Department of Cardiology, Soroka University Medical Center, Beersheva, Israel; ^2^ Faculty of Health Sciences, Ben Gurion University of the Negev, Beersheva, Israel; ^3^ Lev Leviev Heart and Vascular Center, Sheba Medical Center, Tel Hashomer, Israel; ^4^ Department of Cardiology, Rabin Medical Center, Petah Tikva, Israel

**Keywords:** ventricular arrhythmia, atrial fibrillation, atrioventricular block, acute myocardial infarction, women

## Abstract

**Background:**

Acute myocardial infarction (AMI) complicated by tachyarrhythmias or high-grade atrioventricular block (HAVB) may lead to increased mortality.

**Purpose:**

To evaluate the sex differences in patients with AMI complicated by tachyarrhythmias and HAVB and their associated outcomes.

**Materials and methods:**

We analyzed the incidence rates of arrhythmias following AMI from the Acute Coronary Syndrome Israeli Survey database from 2000 to 2018. We assessed the differences in arrhythmias incidence and the associated mortality risk between men and women.

**Results:**

This cohort of 14,280 consecutive patients included 3,159 (22.1%) women and 11,121 (77.9%) men. Women were less likely to experience early ventricular tachyarrhythmia (VTA), (1.6% vs. 2.3%, *p* = 0.034), but had similar rates of late VTA (2.3% vs. 2.2%, *p* = 0.62). Women were more likely to experience atrial fibrillation (AF) (8.6% vs. 5.0%, *p* < 0.001) and HAVB (3.7% vs. 2.3%, *p* < 0.001). The risk of early VTAs was similar in men and women [adjusted Odds Ratio (aOR) = 0.76, *p* = 0.09], but women had a higher risk of AF (aOR = 1.27, *p* = 0.004) and HAVB (aOR = 1.30, *p* = 0.03). Early [adjusted hazard ratio (aHR) = 2.84, *p* < 0.001] and late VTA (aHR =- 4.59, *p* < 0.001), AF (aHR = 1.52, *p* < 0.001) and HAVB (aHR = 2.83, *p* < 0.001) were associated with increased 30-day mortality. Only late VTA (aHR = 2.14, *p* < 0.001) and AF (aHR = 1.44, *p* = 0.002) remained significant in the post 30 days period.

**Conclusions:**

During AMI women experienced more AF and HAVB but fewer early VTAs than men. Early and late VTAs, AF, and HAVB were associated with increased 30-day mortality. Only late VTA and AF were associated with increased post-30-day mortality.

## Introduction

Women with acute coronary syndrome (ACS) have longer hospitalizations, more in-hospital complications, and increased mortality as compared to men (1–6). Despite significant therapeutic advances over the last decades, acute myocardial infarction (AMI) is frequently complicated by supraventricular and ventricular tachyarrhythmia as well as conduction disturbances which are related to increased morbidity and mortality (7–14).

Early ventricular tachyarrhythmias (within 48 h after AMI) (VTAs) are the most frequent cause of death early after AMI (15, 16). Prior studies from the thrombolytic (17, 18) and percutaneous coronary intervention (PCI) era (19–22) showed a higher risk of long-term death associated with late VTAs (after 48 h from AMI) but not with early onset VTAs.

Atrial fibrillation (AF) often complicates AMI with an incidence between 6% and 21% (23–28). AF may cause further impairment of coronary circulation and left ventricular function and it remains a strong predictor of mortality (28, 29).

Despite the implementation of PCI and the decreasing occurrence of HAVB in patients hospitalized for AMI, the occurrence of HAVB in MI remains significant with reported incidence rates between 2.7% overall in AMI patients (30) and 3.5% in ST-elevation myocardial infarction (STEMI) patients treated with primary PCI (31). Moreover, this complication continues to have serious adverse prognostic implications also in the PCI era (30, 31).

Most of the studies regarding arrhythmias in the setting of AMI were conducted before the PCI era, and most of them did not specifically investigate differences between men and women in terms of incidence rates and outcomes. In the current study, we aimed to investigate the incidence and outcome of tachyarrhythmias and HAVB in women as compared to men in a large national registry of unselected consecutive patients with AMI.

## Materials and methods

We collected data from the Acute Coronary Syndrome Israeli Survey (ACSIS) conducted between 2000 and 2018. Briefly, the ACSIS Registry, a 2-month nationwide survey conducted biennially for more than 20 years, prospectively collects data from all consecutive ACS admissions in all 25 coronary care units in Israel. Patient management is left to the discretion of the attending physicians. Discharge diagnoses were recorded as determined by the attending physicians based on clinical, electrocardiographic, echocardiographic, and biomarker criteria. Dedicated study personnel recorded demographic, historical, and clinical data, including medical management, on prespecified study forms. The Central Data Coordinating Center was responsible for collecting all case report forms, and the Israel Heart Society was responsible for keeping the survey database. Thirty-day outcomes and 1-year mortality were ascertained by hospital chart review, telephone contact, and use of the Israeli National Population Registry. For the present study we used data solely from patients with STEMI or non-ST-elevation myocardial infarction (NSTEMI) This register-based analysis of pre-existing data was conducted according to the principles expressed in the Declaration of Helsinki and ethics committees approved the ACSIS in each of the participating centers. All patients provided written informed consent for data collection and subsequent analysis. Endpoints were prespecified by the ACSIS steering committee. The attending physician made the diagnosis of AMI using all available data based on the Universal Definition of Myocardial Infarction (32–34).

We compared the specific incidence rates of in-hospital arrhythmias between men and women. The arrhythmias of interest included: new-onset AF, HAVB, or sustained VTAs [sustained ventricular tachycardia (VT)/ventricular fibrillation (VF)].

New onset AF was defined as the occurrence of AF as an in-hospital complication in the absence of known previous AF at baseline. HAVB was defined if either complete AV block or Mobitz type II second grade AV block occurred during the index hospitalization.

Sustained VT during the index hospitalization was defined as either lasting more than 30 s or requiring termination earlier due to instability. Sustained ventricular arrhythmia (VT/VF) was further categorized as early (within 48 h from MI) or late (more than 48 h). For every subtype of these arrhythmias (AF, VT/VF, HAVB), the outcomes of patients were compared between those who experienced the specific arrhythmia during the index hospitalization compared to those without it in men and women.

### Statistical analysis

Baseline demographic and clinical characteristics are presented as numbers and percentages for categorical variables and median values and interquartile ranges or mean (SD) values for continuous variables. Categorical variables were compared using the *χ*^2^ test, and continuous variables were compared using the Wilcoxon rank sum test or the t-test as appropriate. To obtain odds ratios (ORs) with a 95% confidence interval (CI) for the occurrence of every specific arrhythmia (AF, VTAs, and HAVB) in women vs. men (reference), univariate and multivariable logistic regression models were performed. Covariate adjustment with the propensity score was performed.; the propensity score was built using logistic regression model (one for each of the dependent variables: AF, VTAs, HAVB) and evaluates the probability for the dependent variable, including the following prespecified covariates: age, chronic renal failure at baseline, prior myocardial infarction (MI), congestive heart failure (CHF) at baseline, beta-blockers (BB) treatment at baseline, STEMI diagnosis, left ventricular ejection fraction (LVEF), Killip class at presentation, hypertension, diabetes and peak CK. Multiple imputation was used for missing values in the included covariates. Survival curves were presented to assess the relationship between gender and 1-year mortality, and the Kaplan-Meier pairwise log-rank tests with Holm's *p*-value adjustment were used. The associations between the occurrence of every specific in-hospital arrhythmia (AF, VTAs, HAVB) and all-cause mortality (30-day mortality, 1-year mortality, 30-day to 1-year mortality) were evaluated in the total cohort and separately among men and women using multivariable Cox proportional hazards models, adjusted for propensity score as described above. Interaction between gender and each of the in-hospital arrhythmias was assessed. All tests were conducted at a two-sided overall 5% significance level (*p *= 0.05). All analyses were performed using R statistical software (R-studio, V.4.0.3, Vienna, Austria).

## Results

### Patient characteristics

Among 14,280 consecutive patients with AMI (93% had type 1 MI), 3,159 were (22.1%) women and 11,121 (77.9%) men. Women were older (72 vs. 61 years, *p* < 0.001) and were more likely to have diabetes, hypertension dyslipidemia, CHF, and a history of cerebrovascular disease. However, women were less likely to have prior MI, previous PCI or coronary artery bypass graft (CABG). Moreover, women were more likely to be treated with BB, calcium channels blockers (CCB), angiotensin-converting enzyme inhibitors (ACEI), angiotensin receptors blockers (ARB), statins and diuretics. There were more women with LVEF below 40% as compared to men. Women were less likely to present with STEMI but were more likely to present with pulmonary edema and cardiogenic shock (Table 1).

**Table 1:** Baseline and admission characteristics of patients.

	Overall (14,280)	Women (3,159)	Men (11,121)	*p*
Age, years [IQR])	64.00 [54.00, 74.00]	72.00 [62.00, 80.00]	61.00 [53.00, 71.00]	<0.001
BMI (kg/m^2^), (IQR)	26.89 [24.49, 29.94]	27.34 [24.22, 31.11]	26.81 [24.51, 29.68]	<0.001
Dyslipidemia	9,004 (63.4)	2,089 (66.4)	6,915 (62.5)	<0.001
Hypertension	8,291 (58.2)	2,298 (72.9)	5,993 (54.1)	<0.001
Smoker	5,603 (39.4)	637 (20.3)	4,966 (44.9)	<0.001
Diabetes mellitus	5,061 (35.5)	1,350 (42.8)	3,711 (33.4)	<0.001
Family history of CAD	3,409 (25.7)	566 (19.6)	2,843 (27.5)	<0.001
Prior MI	4,188 (29.4)	769 (24.4)	3,419 (30.8)	<0.001
Prior CABG	1,200 (8.4)	179 (5.7)	1,021 (9.2)	<0.001
Prior PCI	3,600 (25.3)	571 (18.1)	3,029 (27.3)	<0.001
Chronic renal failure	1,562 (11.0)	369 (11.7)	1,193 (10.8)	0.13
PVD	1,172 (8.2)	233 (7.4)	939 (8.5)	0.058
Prior CVA/TIA	1,159 (8.1)	334 (10.6)	825 (7.4)	<0.001
History of CHF	1,117 (7.8)	320 (10.1)	797 (7.2)	<0.001
Prior medications				
Aspirin	5,515 (44.1)	1,258 (46.0)	4,257 (43.6)	0.025
Clopidogrel	983 (8.0)	197 (7.3)	786 (8.2)	0.13
ACE-I	2,584 (29.6)	655 (34.4)	1,929 (28.3)	<0.001
ARB	1,017 (12.1)	316 (17.1)	701 (10.7)	<0.001
Beta-blockers	4,172 (34.2)	1,137 (42.3)	3,035 (32.0)	<0.001
Statins	5,219 (44.2)	1,285 (49.0)	3,934 (42.8)	<0.001
CCB	2,450 (20.8)	758 (29.0)	1,692 (18.5)	<0.001
Diuretics	1,688 (16.8)	594 (26.5)	1,094 (14.0)	<0.001
Presentation and management				
STEMI	7,701 (53.9)	1,601 (50.7)	6,100 (54.9)	<0.001
Admission Killip class				
I	11,580 (83.2)	2,329 (75.4)	9,251 (85.4)	<0.001
II	1,341 (9.6)	412 (13.3)	929 (8.6)	
III	731 (5.3)	274 (8.9)	457 (4.2)	
IV	270 (1.9)	74 (2.4)	196 (1.8)	
LVEF (%)				
>50%	4,766 (41.1)	1,043 (40.5)	3,723 (41.3)	0.014
40%–50%	3,585 (30.9)	751 (29.2)	2,834 (31.4)	
30%–40%	2,242 (19.3)	535 (20.8)	1,707 (18.9)	
<30%	1,002 (8.6)	247 (9.6)	755 (8.4)	
MR moderate +	297 (2.1)	109 (3.5)	188 (1.7)	<0.001
VSR	21 (0.1)	11 (0.3)	10 (0.1)	0.002
Number of vessels diseased per coronary angiogram				
None	418 (4.4)	131 (6.7)	287 (3.8)	<0.001
1 vessel	3,150 (32.9)	684 (35.1)	2,466 (32.3)	
2 vessels	3,056 (31.9)	580 (29.8)	2,476 (32.5)	
3 vessels	2,946 (30.8)	552 (28.4)	2,394 (31.4)	
Reperfusion therapy				
PCI (total PCI in CCU)	9,014 (63.1)	1,744 (55.2)	7,270 (65.4)	<0.001
CABG (in-hospital)	613 (4.3)	122 (3.9)	491 (4.4)	0.19
Laboratory tests				
Peak troponin T elevated	4,969 (88.5)	1,078 (88.1)	3,891 (88.6)	0.71
Peak troponin I elevated	5,424 (88.4)	1,213 (88.7)	4,211 (88.3)	0.70
Peak CK [IQR])	446 [167.0, 1,189.75]	385 [139.50, 1,030.50]	466 [177.0, 1,238.50]	<0.001
Creatinine (mg/dl)-(IQR)	1 (0.86–1.23)	0.90 (0.74–1.20)	1 (0.9–1.24)	<0.001

ACE-I, angiotensin converting enzymes inhibitors; ARB, angiotensin receptor blocker; BMI, body mass index; CABG, coronary artery bypass graft; CAD, coronary artery disease; CCB, calcium channels blockers; CHF, congestive heart failure; CVA, cerebrovascular accident; LVEF, left ventricular ejection fraction; MI, myocardial infarction; MR, mitral regurgitation; PCI, percutaneous coronary intervention; PVD, peripheral vascular disease; SD, standard deviation; STEMI, ST elevation myocardial infarction; TIA, transient ischemic attack; VSR, ventricular septal rupture.

### In hospital arrhythmias

#### The risk of ventricular arrhythmia in women vs. men

Overall, 306 (2.1%) patients presented with early VTAs, and 316 (2.2%) patients experienced late VTAs (Table 2). Men were more likely to develop early VTA (2.1% vs. 1.6%, *p* = 0.034) but the rate of late VTA was similar in men and women (2.3% vs. 2.2%, *p* = 0.62). After multivariate adjustment, the risk of early VTA [adjusted OR (aOR) 0.76, CI (0.56, 1.03), *p* = 0.09] and late VTA [aOR 1.0, CI (0.76, 1.30), *p* = 0.99] was similar in men and women (Table 2). The aOR of the propensity score is 3.30, 95% CI (2.69, 4.09), *p* < 0.001 for early VTA and 3.84, 95% CI (2.88, 5.08), *p* < 0.001 for late VTA.

**Table 2:** Crude rates and multivariable-adjusted odds ratios of in-hospital arrhythmias in women as compared to men.

In-hospital arrhythmias	Overall (*n* = 14,280)	Women (*n* = 3,159)	Men (*n* = 1,112)	*p*	Adjusted OR^a^ (95% CI)	*p*
Early VTA, *n* (%)	306 (2.1)	52 (1.6)	254 (2.3)	0.034	0.76 (0.56,1.03)	0.09
Late VTA, *n* (%)	316 (2.2)	74 (2.3)	242 (2.2)	0.62	1.0 (0.76,1.30)	0.99
New onset AF, *n* (%)	825 (5.8)	272 (8.6)	553 (5.0)	<0.001	1.27 (1.08,1.49)	0.004
HAVB, *n* (%)	372 (2.6)	116 (3.7)	256 (2.3)	<0.001	1.30 (1.03,1.63)	0.03

AF, atrial fibrillation; HAVB, high degree atrioventricular block; OR, odds ratio; VTA, ventricular tachyarrhythmia.

^a^ Adjusted for propensity score including the following prespecified covariates: age, chronic renal failure at baseline, prior myocardial infarction (MI), congestive heart failure (CHF) at baseline, beta-blockers (BB) treatment at baseline, STEMI diagnosis, left ventricular ejection fraction (LVEF), Killip class at presentation, hypertension, diabetes and peak CK.

#### The risk of atrial fibrillation in women vs. men

Overall, 825 (5.8%) patients had new-onset AF during the index hospitalization. The rate of new-onset AF was higher in women than in men (8.6% vs. 5%, *p* < 0.001). Compared to men, women were at higher risk of incident AF after multivariate analysis [aOR 1.27, CI (1.08, 1.49), *p* = 0.004] (Table 2). The aOR of the propensity score is 2.60, 95% CI (2.39, 2.82), *p* < 0.001.

#### The risk of AV block in women vs. men

HAVB was observed in 327 patients (2.6%), and women were more likely to experience HAVB during the index hospitalization as compared to men (3.7% vs. 2.3%, *p* < 0.001). Women were at higher risk to experience HAVB after multivariate analysis (aOR 1.30, CI (1.03, 1.63, *p* = 0.03) (Table 2). The aOR of the propensity score is 8.34, 95% CI (6.15, 11.24), *p* < 0.001.

### Mortality

One year after AMI, 1,472 (10.6%) deaths occurred. The mortality rate was 8.9% in men and 16% in women, *p* < 0.001 (Table 3).

#### The association of ventricular arrhythmia with mortality risk

Early VTAs were associated with increased 1-year mortality in the total cohort [adjusted HR (aHR) 2.19, CI (1.71, 2.80) *p* < 0.001]. However, this was driven by increased mortality risk at 30-days [aHR 2.84, CI (2.14, 3.77), *p* < 0.001] with no increased risk of death in the post 30 days period after AMI [aHR 1.24, CI (0.68, 2.26), *p* = 0.50]. This association was similar in men and women, as outlined in Table 3.

**Table 3:** Association between in-hospital arrhythmias and mortality in women and men.

	In all cohort adjusted HR (95% CI)	*p*	In women adjusted HR (95% CI)	*p*	In men adjusted HR (95% CI)	*p*
Early VTA						
30-day	2.84 (2.14, 3.77)	<0.001	2.51 (1.46, 4.32)	<0.001	3.02 (2.16, 4.21)	<0.001
1-year	2.19 (1.71, 2.82)	<0.001	1.91 (1.15, 3.17)	0.02	2.30 (1.72, 3.08)	<0.001
30-day to 1-year	1.24 (0.68, 2.26)	0.50	0.45 (0.06, 3.25)	0.44	1.46 (0.77, 2.75)	0.25
Late VTA						
30-day	4.59 (3.70, 5.70)	<0.001	4.57 (3.17, 6.60)	<0.001	4.66 (3.57, 6.10)	<0.001
1-year	3.73 (3.11, 4.48)	<0.001	3.87 (2.79, 5.36)	<0.001	3.68 (2.95, 4.59)	<0.001
30-day to 1-year	2.14 (1.48, 3.09)	<0.001	2.22 (1.04, 4.76)	0.04	2.16 (1.42, 3.28)	<0.001
AF						
30-day	1.52 (1.25, 1.86)	<0.001	1.17 (0.85, 1.62)	0.35	1.83 (1.42, 2.34)	<0.001
1-year	1.49 (1.28, 1.73)	<0.001	1.34 (1.05, 1.71)	0.02	1.64 (1.36, 1.98)	<0.001
30-day to 1-year	1.44 (1.15, 1.82)	0.002	1.61 (1.11, 2.34)	0.02	1.46 (1.09, 1.94)	0.02
HAVB						
30-day	2.83 (2.24, 3.58)	<0.001	2.75 (1.91, 3.96)	<0.001	2.78 (2.04, 3.78)	<0.001
1-year	2.29 (1.88, 2.79)	<0.001	2.26 (1.65, 3.09)	<0.001	2.31 (1.79, 2.98)	<0.001
30-day to 1-year	1.47 (1.00, 2.15)	0.05	1.55 (0.81, 2.95)	=0.19	1.53 (0.95, 2.45)	0.08

AF, atrial fibrillation; HAVB, high degree atrioventricular block; CI, confidence interval; HR, hazard ratio; VF, ventricular fibrillation; VT, ventricular tachycardia.

**Figure 1:**
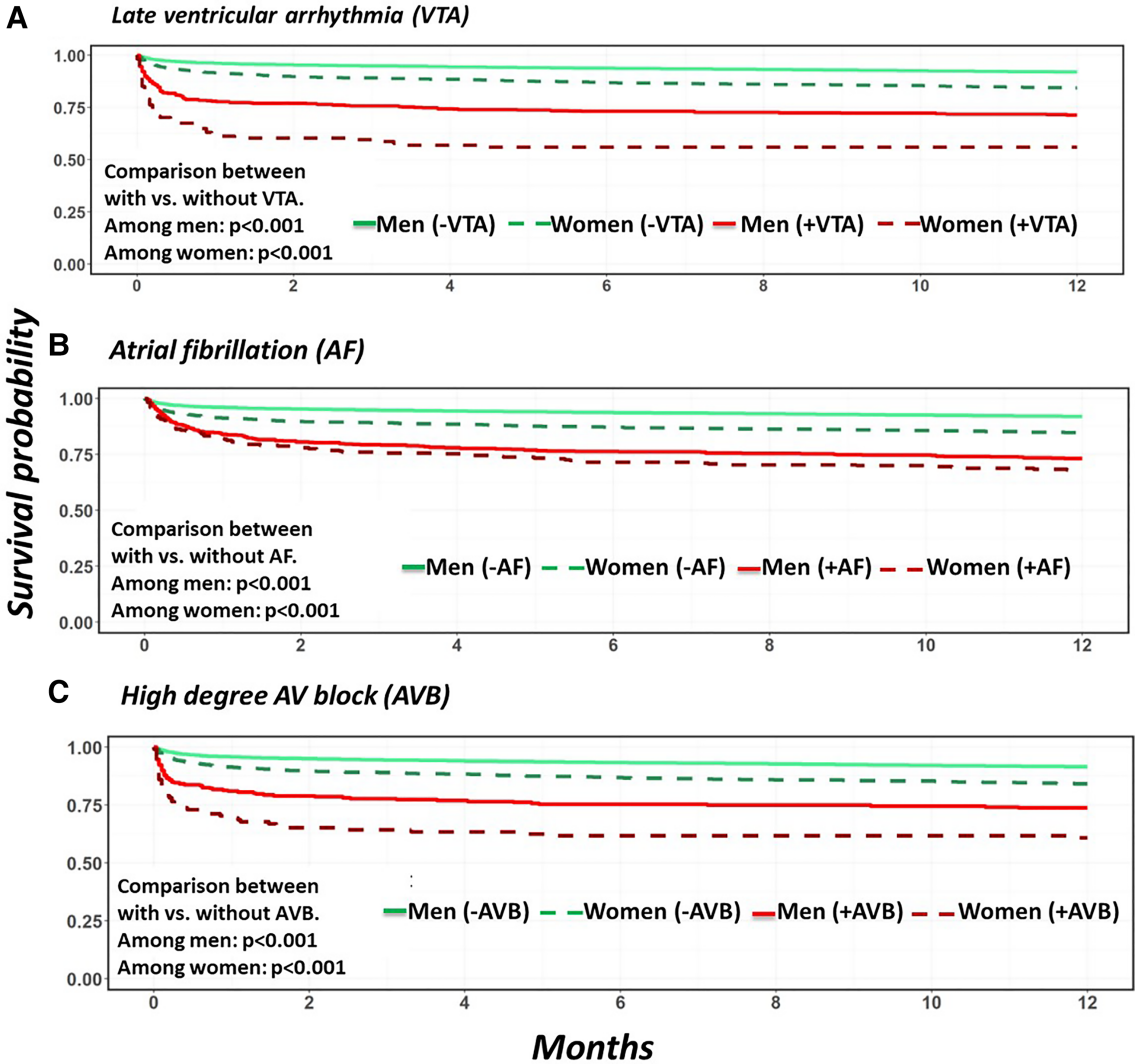
Unadjusted patients’ survival probabilities comparing patients with and without arrhythmia stratified by sex. (**A**) Late sustained ventricular arrhythmias. (**B**) Atrial fibrillation. (**C**) High degree AV block.

Overall, the occurrence of late VTA was found to be associated with increased mortality risk at 30 days [aHR 4.59, CI (3.70, 5.70), *p* < 0.001] and at 1 year [aHR 3.73, CI (3.11, 4.48), *p* < 0.01] including the post 30 days period after MI [aHR 2.14, CI (1.48, 3.09), *p* < 0.001]. The association between late VTAs and mortality was similar and significant in both men and women (Table 3, Figure 1A).

#### The association of atrial fibrillation with mortality risk

New onset AF was found to be associated with increased mortality risk at 30 days, [aHR 1.52, CI (1.25, 1.86), *p* < 0.001], 1 year [aHR 1.49, CI (1.28, 1.73 *p* < 0.01] and in the post 30 days period after AMI [aHR 1.44, CI (1.15, 1.82), *p* = 0.002]. Interestingly, AF was found to be associated with increased 30-day mortality risk in men [aHR 1.83, CI (1.42, 2.34), *p* < 0.001] but not in women [aHR 1.17, CI (0.85, 1.62), *p* = 0.35]. However, the post 30-day mortality risk and therefore the 1-year mortality risks were increased in both men [aHR 1.64, CI (1.36, 1.98), *p* < 0.001] and women [aHR 1.34, CI (1.05, 1.71), *p* = 0.02] who developed AF during their index hospitalization, with a higher relative risk of 1-year mortality in men than women (*p* for interaction <0.001) (Table 3, Figure 1B).

Notably, Patients with AF were more likely to have cardiovascular comorbidities, a history of MI, cerebrovascular disease, and peripheral vascular disease. Patients with AF were more likely to present with multivessel disease, severe left ventricular dysfunction, pulmonary edema, and cardiogenic shock. Bleeding events were higher in patients with AF, and they were less likely to undergo coronary revascularization compared to their counterparts. Patients with AF had a higher incidence of stroke during the index hospitalization and only 22.5% of them were discharged with oral anticoagulation (Supplementary Table S5 in the Supplementary Material).

#### The association of AV block with mortality risk

The occurrence of HAVB was found to be associated with increased 1-year mortality in the total cohort [aHR 2.29, CI (1.88, 2.79), *p* < 0.01]. This was mainly driven by an increased risk of mortality at 30 days [aHR 2.83, CI (2.24, 3.58), *p* < 0.001]. However, the association between HAVB and mortality risk in the post 30 days period after MI did not reach statistical significance [aHR 1.47, CI (1.00, 2.15), *p* = 0.05] (Table 3, Figure 1C).

Analysis for patients with STEMI and non-STEMI are provided in the Supplementary Tables S1–S4.

## Discussion

The main findings of this real-world study of patients with AMI are: (1) Men were more likely than women to experience sustained early VTAs but the incidence of late VTAs was similar in men and women. (2) Women were more likely to experience AF and HAVB during their index hospitalization. (3) Early and late VTAs, AF, and HAVB were all associated with increased 30-day mortality whereas only late VTA and AF were associated with increased mortality in the post 30-day period. Early VTAs and HAVB were not associated with excess risk of death after hospital stay.

### Ventricular arrhythmia

In the current study, VTAs occurred in 4.3% (2.1% early VTAs and 2.2% late VTAs) of patients, with early onset (<48 h) VTA less prevalent in women. Both early and late VTA were associated with short-term mortality whereas only late VTAs were associated with long-term mortality. These findings are similar in men and women. Earlier studies have reported incidence rates of VTAs between 6% in patients with AMI undergoing PCI and 10% in patients who received thrombolytic therapy (17, 19). However, these studies included mainly patients presenting with STEMI. In a previous report from our group, we demonstrated that VTAs occurred in 3.8% of patients [2.1% early (≤48 h) and 1.7% late (>48 h) VTA], similar to the present but somewhat later cohort (20). Prior studies from the thrombolytic era showed that women and men appear to be at similar risk for developing VTAs after AMI (17, 21). The impact of VTAs on the short and long-term prognosis of patients with AMI has been debated over the years. Sustained VTAs >48 h after index MI have been associated with increased mortality risk (35). However, the relationship between early VTAs and mortality remains controversial. Some studies reported that both early and late VTAs were found to be associated with increased late mortality, with late arrhythmias carrying a worse prognosis (17, 19). Others have suggested that the early post-MI sustained VTAs may be associated with increased short-term mortality but without increased risk over the long term (20, 22). In a very recent data analysis from the FAST-MI (French Registry of Acute ST-Elevation or Non-ST-Elevation Myocardial Infarction) program, 2.5% of patients developed VF. Similarly, women had a lower risk of developing VF during AMI compared with men, and the risk of death associated with VF was similar in men and women (36). However, the aforementioned study included only STEMI patients and the arrhythmia of interest was VF compared to combined VF or sustained VT in our study.

### Atrial fibrillation

We found that 5.8% of the patients had new-onset AF with women's predominance. AF was found to be associated with decreased survival which was more pronounced in men as compared to women. In the APEX-AMI trial of 5,745 STEMI patients treated with primary PCI, 6.3% of patients developed new-onset AF, and it was associated with heart failure, cardiogenic shock, stroke, and increased 90-day mortality (28). Similarly, we found that patients with AF were more likely to present with multivessel disease, severe left ventricular dysfunction, pulmonary edema, and cardiogenic shock as well as a higher incidence of stroke during the index hospitalization. However, our study included patients with STEMI and NSTEMI, and the rate of new-onset AF was similar to APEX-AMI. In a recent study of 6,228 patients with AMI who underwent PCI, the rate of newly diagnosed AF was slightly higher (7.9%) and it was associated with an increased risk of death (37). Interestingly, new-onset AF was found to be associated with increased 1-year mortality risk in both men and women in the present study. Meta-analysis of 30 cohort studies that reported sex specific associations between AF (not MI-related) and all-cause mortality showed that AF is a stronger marker for death in women compared with men (38). However, data on specific sex related differences in the incidence and outcomes of AF complicating AMI are scarce. Thus, our findings prompt the need for further studies assessing the prognostic importance of AF and AF management in men as compared to women.

### AV block

In our study, the overall incidence rate of HAVB was 2.6%. The occurrence of HAVB was higher in women and was associated with increased risk of death similarly in men and women. Data from the thrombolytic era suggest that 6.9% of patients hospitalized with STEMI develop HAVB, and it is associated with increased 30-day and 1-year mortality. Moreover, women were more likely to develop HAVB than men (39). Later report from the PCI era showed that the incidence of HAVB among STEMI patients has decreased (3.2%) with the implementation of primary PCI. Despite this, it remained a severe prognostic factor also in the PCI era. Notably, like in the present study, female gender was found to be predictive of developing HAVB in STEMI patients (31). More recent analysis from our group that included STEMI and NSTEMI patients suggests a similar rate (2.7%) of HAVB that decreases over time and is associated with increased 30-day and 1-year mortality (30). To the best of our knowledge, no previous studies specifically investigated differences in short and long-term outcomes of HAVB between men and women. Interestingly, a recent cohort study of 443 patients with unexplained syncope and bundle branch block showed that, compared to men, women have a lower risk of AV block and need for cardiac pacing. However, only 21% of patients had ischemic heart disease and no patients with AMI were included (40).

### Limitations

Our study has several limitations inherent to the observational, retrospective, nonrandomized design of this study. As in any observational study, we could not exclude residual confounding and associations despite the adjustment for the most clinically relevant variables. Our study focused on in-hospital arrhythmias, and arrhythmias occurring out of the hospital might be under-reported. Moreover, data on temporary or permanent pacing and antiarrhythmic medications were not systematically collected. The main strengths of our study are the large sample size with prospectively collected data in a uniform case report form and standard definitions used in all centers and endpoints that were centrally adjudicated.

In conclusion, in this contemporary cohort of patients with AMI, the incidence rate of early VTA was higher among men whereas the rate of late VTA was similar in men and women. Women experienced more AF and HAVB than men. Early VTA, late VTAs, AF, and HAVB were associated with increased short-term mortality risk whereas only late VTA and AF were associated with increased long-term mortality.

## Data Availability

The raw data supporting the conclusions of this article will be made available by the authors, without undue reservation.
