# Supplementary material for: Sex differences in ventricular arrhythmia, atrial fibrillation and atrioventricular block complicating acute myocardial infarction
Source: Front Cardiovasc Med. 2023 Oct 19;10:1217525. doi: 10.3389/fcvm.2023.1217525 (PMC10620835; doi:10.3389/fcvm.2023.1217525)
Supplement: Supplementary file 1 [file Table1.docx]

Table S1: Crude rates and multivariable-adjusted odds ratios of in-hospital arrhythmias in women as compared to men presenting with ST-elevation myocardial infarction

| *In-hospital arrhythmias* | *Overall*  *(n=7701)* | *Women*  *(n=1601)* | *Men*  *(n=6100)* | *P* | *Adjusted OR*  *(95%CI)* | *P* |
| --- | --- | --- | --- | --- | --- | --- |
| Early VTA, n (%) | 271 (3.5) | 46 (2.9) | 225 (3.7) | 0.13 | 0.84(0.60,1.15) | 0.30 |
| Late VTA, n (%) | 221 (2.9) | 53 (3.3) | 168 (2.8) | 0.27 | 0.96(0.69,1.32) | 0.80 |
| AF, n (%) | 472 (6.1) | 144 (9.0) | 328 (5.4) | <0.001 | 1.08(0.86,1.34) | \|  \|  \| \| --- \| --- \| \|  \|  \|   0.51 |
| HAVB, n (%) | 297 (3.9) | 92 (5.8) | 205 (3.4) | <0.001 | 1.40(1.07,1.81) | 0.02 |

AF-atrial fibrillation, HAVB- high degree atrioventricular block, OR- odds ratio, VTA- ventricular tachyarrhythmia.

Table S2. Association between in-hospital arrhythmias and mortality in women and men presenting with ST-elevation myocardial infarction

| P | In men Adjusted HR (95%CI) | P | In women Adjusted HR (95%CI) | P | In all cohort Adjusted HR (95%CI) |  |
| --- | --- | --- | --- | --- | --- | --- |
| <0.001  <0.001  0.85 | 2.67(1.84,3.87)  2.06(1.48,2.88)  1.08(0.48,2.46) | 0.07  0.20  0.45 | 1.77(0.95,3.30)  1.47(0.82,2.64)  0.47(0.06,3.36) | <0.001  <0.001  0.84 | 2.40(1.75,3.30)  1.90(1.43,2.54)  0.92(0.43,1.96) | Early VTA  30-day  1-yr  30-day to 1-yr |
| <0.001  <0.001  0.03 | 4.82(3.52,6.60)  3.81(2.90,5.02)  2.02(1.11,3.67) | <0.001  <0.001  0.03 | 4.51(3.01,6.77)  4.17(2.89,6.03)  2.85(1.13,7.19) | <0.001  <0.001 0.004 | 4.67(3.65,5.97) 3.95(3.17,4.91) 2.11(1.27,3.49) | Late VTA  30-day  1-yr  30-day to 1-yr |
| 0.002  <0.001  0.004 | 1.67(1.22, 2.29)  1.69(1.32,2.17)   \|  \| \| --- \|   1.80(1.21,2.67) | 0.57  0.33  0.27 | 1.13(0.75,1.68)  1.19(0.85, 1.66) 1.43(0.76, 2.68) | 0.005 <0.001  0.02 | 1.43(1.12,1.83)  1.46(1.20, 1.79)  1.53(1.08,2.16) | AF  30-day  1-yr  30-day to 1-yr |
| <0.001 <0.001 0.09 | 3.03(2.16,4.25)  2.51(1.87,3.35)  1.67(0.93,3.00) | <0.001  <0.001  0.82 | 2.60(1.75,3.89)  2.17(1.52,3.11)  1.10(0.48,2.56) | <0.001  <0.001  0.04 | 3.05(2.37,3.94)  2.60(2.08,3.25)  1.66(1.03,2.68) | HAVB  30-day  1-yr  30-day to 1-yr |

AF- atrial fibrillation, HAVB- high degree atrioventricular block, CI-confidence interval, HR- hazard ratio, VF- ventricular fibrillation, VT- ventricular tachycardia.

Table S3: Crude rates and multivariable-adjusted odds ratios of in-hospital arrhythmias in women as compared to men presenting with Non-ST-elevation myocardial infarction

| *In-hospital arrhythmias* | *Overall*  *(n=6579)* | *Women*  *(n=1558)* | *Men*  *(n=5021)* | *P* | *Adjusted OR*  *(95%CI)* | *P* |
| --- | --- | --- | --- | --- | --- | --- |
| Early VTA, n (%) | 35(0.5) | 6 (0.4) | 29 (0.6) | 0.48 | 0.68(0.25,1.53) | 0.39 |
| Late VTA, n (%) | 95 (1.4) | 21 (1.3) | 74 (1.5) | 0.81 | 0.80(0.48,1.29) | 0.39 |
| AF, n (%) | 353 (5.4) | 128 (8.2) | 225 (4.5) | <0.001 | 1.42(1.12,1.80) | \|  \|  \| \| --- \| --- \| \|  \|  \|   0.004 |
| HAVB, n (%) | 75 (1.1) | 24 (1.5) | 51 (1.0) | 0.12 | 1.18(0.70,1.94) | 0.52 |

AF-atrial fibrillation, HAVB- high degree atrioventricular block, OR- odds ratio, VTA- ventricular tachyarrhythmia.

Table S4. Association between in-hospital arrhythmias and mortality in women and men presenting with Non-ST-elevation myocardial infarction

| P | In men Adjusted HR (95%CI) | P | In women Adjusted HR (95%CI) | P | In all cohort Adjusted HR (95%CI) |  |
| --- | --- | --- | --- | --- | --- | --- |
| <0.001  <0.001  0.08 | 4.74(2.20,10.22)3.65(2.00,6.67) 2.52(0.90,7.01) | <0.001<0.001 | 11.57(3.62,37.00)8.92(3.23, 24.59)  NA^*^ | <0.001  <0.001  0.08 | 7.19(3.97,13.02) 4.86(2.94, 8.06) 2.50(0.92, 6.82) | Early VTA  30-day  1-yr  30-day to 1-yr |
| <0.001  <0.001  0.004 | 3.56(2.11,6.02)  2.84(1.93, 4.20)  2.41(1.34, 4.32) | 0.002  0.004  0.59 | 3.99(1.71, 9.32)  2.91(1.43, 5.93)  1.48(0.37, 6.02) | <0.001 <0.001 0.005 | 3.87(2.48, 6.03) 2.95(2.10, 4.15) 2.19(1.28, 3.75) | Late VTA  30-day  1-yr  30-day to 1-yr |
| 0.0002  <0.001 0.27 | 2.18(1.46, 3.25)  1.63(1.23, 2.16)  1.26(0.84, 1.90) | 0.38  0.01 0.02 | 1.29(0.73, 2.27)  1.60(1.12, 2.27)  1.79(1.13, 2.82) | <0.001  <0.001 0.04 | 1.78(1.29, 2.47)  1.57(1.26, 1.96)  1.39(1.02, 1.88) | AF  30-day  1-yr  30-day to 1-yr |
| 0.06 0.06 0.52 | 2.22(0.98, 5.03) 1.76(0.99, 3.14) 1.31(0.58, 2.96) | 0.01  0.01 0.19 | 3.10(1.31, 7.33)  2.38(1.23, 4.59)  1.98(0.72, 5.49) | 0.01  0.02 0.38 | 2.26(1.24, 4.10)  1.72(1.11, 2.66)  1.34(0.70, 2.55) | HAVB  30-day  1-yr  30-day to 1-yr |

*Low event rate

AF- atrial fibrillation, HAVB- high degree atrioventricular block, CI-confidence interval, HR- hazard ratio, VF- ventricular fibrillation, VT- ventricular tachycardia.

Table S5: Baseline and admission characteristics of patients with atrial fibrillation (AF)compared to those without AF.

| P | AF  825 | Without AF  13451 | |  |
| --- | --- | --- | --- | --- |
| <0.001 | 75.0 [66.0, 81.0] | 63.0 [54.0, 73.0] | | Age, years (median [IQR]) |
| <0.001 | 553 (67.0) | 10565 (78.5) | | Men |
| <0.001 | 560 (68.4) | 7727 (57.6) | | Hypertension |
| 0.003 | 331 (40.4) | 4728 (35.2) | | Diabetes mellitus |
| 0.007 | 276 (33.7) | 3911 (29.1) | | Prior MI |
| <0.001 | 179 (21.8) | 1382 (10.3) | | Chronic renal failure |
| <0.001 | 109 (13.3) | 1062 (7.9) | | PVD |
| 0.002 | 90 (11.0) | 1067 (8.0) | | s/p CVA/TIA |
| <0.001 | 128 (15.6) | 986 (7.3) | | History of CHF |
| Presentation | | | | |
| 0.056 | 472 (57.2) | 7228 (53.7) | | STEMI |
| <0.001 | 146 (20.7) | 855 (7.9) | | LVEF<30% |
| Number of vessels diseased | | | | |
| <0.001 | 109 (24.7) | 3041 (33.3) | | 1 vessel |
|  | 143 (32.4) | 2913 (31.9) | | 2 vessels |
|  | 175 (39.7) | 2768 (30.3) | | 3 vessels |
| Reperfusion therapy | | | | |
| <0.001 | 427 (51.8) | 8585 (63.8) | | PCI (total PCI in CCU) |
| 0.84 | 37 (4.5) | 576 (4.3) | | CABG (total CABG in CCU) |
| In-hospital complications | | | | |
| <0.001 | 206 (25.1) | 812 (6.0) | Pulmonary edema | |
| <0.001 | 117 (14.3) | 451 (3.4) | | Cardiogenic shock |
| <0.001 | 63 (7.7) | 234 (1.7) | | MR moderate - severe |
| <0.001 | 24 (2.9) | 74 (0.6) | | Stroke |
| <0.001 | 220 (26.8) | 698 (5.2) | | Acute renal failure |
| <0.001 | 40 (4.9) | 185 (1.4) | | Bleeding |
|  |  |  | | **Treatment at discharge** |
| <0.001 | 168 (22.5) | 620 (5.0) | | Oral Anticoagulation |

CABG-coronary artery bypass graft, CHF- congestive heart failure, CVA- cerebrovascular accident, LVEF-left ventricular ejection fraction, MI-myocardial infarction, MR- mitral regurgitation, PCI- percutaneous coronary intervention, PVD- peripheral vascular disease, STEMI- ST elevation myocardial infarction, TIA- transient ischemic attack.
